# Systematic Review of Social Marketing as a Behavior Change Agent in Salt Reduction

**DOI:** 10.5334/gh.1478

**Published:** 2025-10-24

**Authors:** Silvia Sommariva, Dove Wimbish, Sarah Mayes, Angela Makris, Virginia Liddell, Mahmooda Khaliq

**Affiliations:** 1 College of Public Health, University of South Florida, Tampa, FL, US

**Keywords:** sodium, salt, behavior change, social marketing, health communication, health promotion

## Abstract

The use of social marketing (SM) interventions for salt/sodium reduction has drawn increased attention worldwide. This systematic review investigates the application of social marketing principles to the design, implementation, and evaluation of salt/sodium reduction interventions globally and provides recommendations for future public health practice. Using PRISMA, searches were conducted on PubMed, Web of Science, CINAHL, and PsychInfo, with 51 final studies identified, abstracted, and synthesized using the matrix method. Studies conducted more recently contained a greater number of social marketing benchmark criteria (behavioral focus, formative research, segmentation, exchange, competition, marketing mix, community involvement, and integration). Studies reporting greater success used more benchmark criteria. Community-based initiatives using personalized/localized tactics combined with upstream policy-supported structural measures and management-supported place-based initiatives implemented in hospitals, workplaces, and schools were the most self-reported effective interventions. Future salt/sodium reduction initiatives should apply the full social marketing framework to multilevel interventions designed with culturally responsive community-based processes.

## Introduction

Excessive salt/sodium consumption is a global public health concern (1,2). Nearly everywhere around the globe, salt intake exceeds recommended levels; in 181 out of 187 countries, people consume more than the World Health Organization’s recommended target of <2 g of sodium per day (2,3,4). While sodium has an essential function in cellular homeostasis and physiological processes and sodium deficiency can cause severe health complications (5,6), excessive consumption has been associated with significant health risks. High sodium intake contributes to increased blood pressure or hypertension, which is itself a risk factor for cardiovascular disease and stroke (6,7,8,9,10). Studies suggest that a shift toward lower salt consumption could produce significant public health benefits, such as a decrease in the number of deaths due to stroke and ischemic heart disease and an overall increase in survival (11,12,13). A decrease in cases of hypertension, related mortality, and morbidity would also have a positive economic impact in terms of lower health care costs (12,13,14,15,16,17). At least three studies—one in 2013 and two in 2024—have found that reducing salt intake is a cost-effective strategy for preventing cardiovascular diseases (16,17,18).

However, regulating sodium consumption is challenging for several reasons. As a behavior, sodium intake requires constant monitoring, since the prevalence of processed foods has made exposure to high-sodium products difficult to avoid (19). To reduce sodium consumption, individuals can adopt behavioral changes on multiple levels (20): from purchasing behavior (e.g., reading labels, selecting low-sodium products at restaurants) to cooking practices (e.g., cooking low-sodium recipes, removing saltshakers from the table). Sources of sodium also differ by context. For example, high-income communities consume most of their sodium from processed and packaged foods, whereas sodium intake in low- and middle-income communities tends to occur through discretionary salt use (21). The habitual and unconscious nature of these behaviors makes sodium reduction a challenging issue to address (20). Moreover, sodium consumption is deeply connected to policy environments, industry regulation, and food culture, which vary across geographical regions and community/family contexts (20,22,23). In recent years, growing policy momentum around salt substitution has shifted the focus from individual-level clinical interventions to population-level prevention strategies as a more effective means of reducing salt consumption (24). This paradigm shift was recognized in the WHO’s 2025 guidelines on salt substitution, which recommend replacing 100% sodium chloride (NaCl) at least in part with potassium enriched salt (KCl) (4,24).

This call for systemic sodium-reduction strategies in food environments positions social marketing as a natural partner in global efforts to lower blood pressure and reduce cardiovascular disease (CVD). Historically, social marketing has been applied increasingly as a program-planning framework for dietary behavior change, and has proven effective at fostering change at both the individual and policy levels (25,26,27,28,29,30,31). In this review, social marketing is defined as the application of marketing concepts to influence behaviors for social good. It is characterized by benchmark criteria such as a focus on behavior change, audience segmentation, formative research, motivational exchanges that appeal to the target audience, analysis of competing behaviors, and marketing mix (price, promotion, product, and place), as well as community involvement, and integration of upstream and downstream strategies (Table 1; 32,33,34,35).

**Table 1:** Social marketing benchmark criteria.


SCORECARD ELEMENT #	CRITERIA	DEFINITION

1	Behavioral focus	The study aims to change behavior

2	Segmentation	The intervention addressed a specific population group and provided a rationale for the targeting/selection strategy

3	Formative research	Research on the priority population was conducted prior to the definition of the intervention

4	Exchange	The intervention creates motivational exchanges that are attractive to the priority population

5	Competition	There is consideration of competing behaviors that the priority population may be inclined to adopt

6	Marketing mix	Use of at least one of the 4Ps derived from marketing strategy

7	Community-involvement	Community plays a role in the design, implementation, and evaluation of the program

8	Integration	The intervention is positioned within broader policy-program efforts that address the specific public health issue, and the design takes into consideration stakeholders’ activities


Since the early 2000s, the World Health Organization (WHO) and the Pan American Health Organization (PAHO) have collaborated with country-level experts to identify solutions to address excess sodium consumption, study the potential of evidence-based tools, and build capacity for their use (1,22,28,36,37,38,39,40). Recommended interventions include sodium reduction in food products, labeling, and mass media campaigns—measures that can create sustainable structural change. The WHO’s Global Database on the Implementation of Food and Nutrition Action (GIFNA) tracks policy implementation worldwide. As of June 13, 2025, only 28.1% of the world’s population lives in countries with mandatory sodium reduction measures (41).

In two reports (published in 2017 and 2018) and an update released in 2024, the WHO cited social marketing as a ‘best buy’ for tackling non-communicable diseases because it reduces risk factors and promotes healthy food environments. While these documents also recommend Behavior Change Communication (BCC) and mass media campaigns, social marketing differs in that it applies marketing techniques to promote sustainable change through perceived benefits (exchange). These reports highlight social marketing’s cost-effectiveness and affordability as an evidence-based intervention to reduce premature deaths, illness, and disability, and its ability to deliver the greatest possible health impacts (42,43,44,45). Despite this, a 2018 systematic review found that the WHO’s ‘best buy’ interventions were substantially underutilized (46).

Other systematic reviews have addressed salt/sodium reduction: Bhat et al. (21) reviewed global dietary salt sources; Santos et al. (47) examined global salt reduction initiatives from 2014 to 2019; Carrillo-Larco and Bernabe-Ortiz (48) reviewed salt consumption in Latin America and the Caribbean; and Vargas-Meza et al. (49) analyzed population- and individual-level salt reduction studies (2018–2022) (21,47,48,49). While these reviews included some social marketing interventions, none specifically analyzed the efficacy of social marketing as a behavior-change methodology in salt reduction across its full historical arc. Despite recent calls for systematic reviews of sodium reduction interventions (50), and at least one review suggesting that the number of benchmark criteria influences intervention effectiveness (51), no systematic review has yet examined social marketing salt initiatives from their inception to ascertain trends in self-reported efficacy over time, nor analyzed trends in efficacy relative to benchmark criteria use.

Systematic reviews provide a powerful methodology to synthesize the state of knowledge in a particular field, identify variations and gaps in practice, investigate conflicting results, and guide future research, policy, and practice (52,53,54,55,56). This systematic review attempts to answer the following questions: (1) How has social marketing been used to reduce salt and sodium consumption over time? (2) What trends exist in the use of social marketing benchmark criteria across salt reduction interventions? (3) What correlations exist between the use of social marketing benchmark criteria and the self-reported effectiveness of salt reduction interventions, and (4) What insights can this provide for future research and practice?

## Methods

### Selection criteria

Studies selected for review had to be interventions designed to reduce excessive sodium/salt consumption published in English in a peer-reviewed journal, and had to incorporate at least one social marketing benchmark criterion (Table 1). The minimum requirement for inclusion was a behavioral focus—that is, a short- or long-term goal of reducing salt/sodium consumption or use. We set no limits on publication date, but excluded conference abstracts, policy briefs, book chapters, and letters to editors because of their potential lack of rigor and higher risk of bias, in line with the *Cochrane Handbook for Systematic Reviews of Interventions* (57).

### Data collection

Team members SS, MK, and DW reviewed MeSH terms, salient papers, and consulted subject matter experts to develop a search term strategy around the concepts of salt and behavior change. Search strings included combinations of indexed and loose terms related to salt consumption and reduction such as ‘salt’, ‘sodium’, ‘restrict’, and ‘reduction’, as well as terms related to social marketing, such as ‘social marketing’, ‘behavior change’, ‘health promotion’, and ‘health communication’. Reference lists of the most relevant articles were also screened for additional studies not identified through database searches. Boolean operators ‘AND’ and ‘OR’ were used to combine terms; the ‘NOT’ operator was not used, following Cochrane guidance (58). See *Supplement 1—Full Search Strategy* for a more in-depth description of the search strategy. Using the Preferred Reporting Items for Systematic Reviews and Meta-Analysis, PRISMA (54), we conducted a systematic search on PubMed, Web of Science, CINAHL, and PsycINFO up to January 1, 2022.

This study was deemed exempt from IRB review because no human subjects were involved, in accordance with US Department of Health and Human Services guidelines (45 CFR 46) (59).

### Study selection

**Figure 1:**
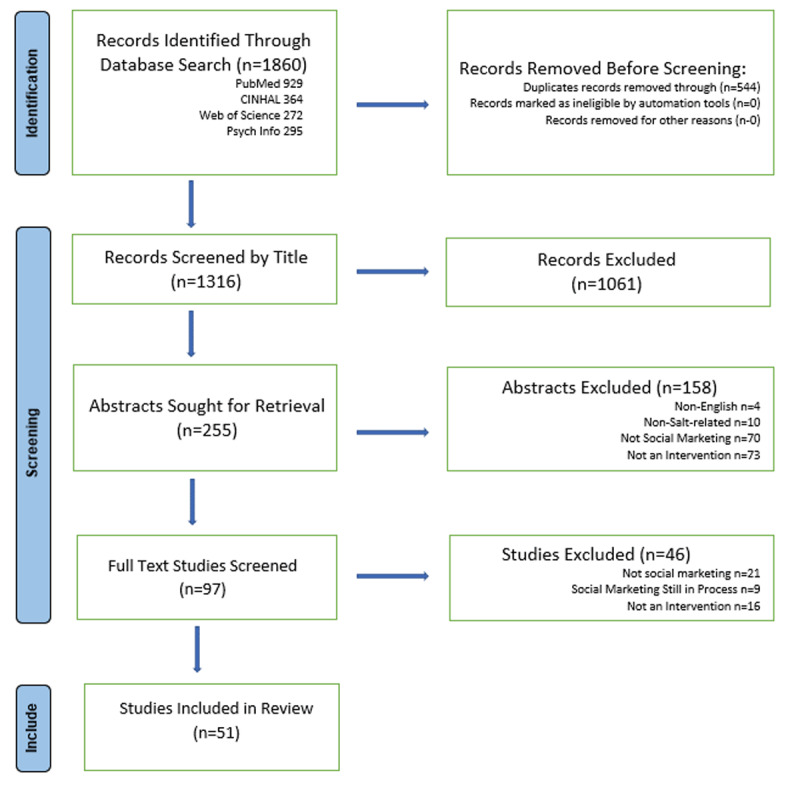
Study selection PRISMA diagram.

We used a team-based, three-step process to conduct this review and to minimize risk of bias. In step one, after jointly selecting databases, refining search terms, and testing search strategies, SS and DW conducted the search, identifying 1860 records (929 from PubMed, 364 from CINHAL, 272 from Web of Science, and 295 from PsycINFO). After removing 544 duplicates, 1316 unique records remained. In step two, SS, DW, SM, AM, and VL, graduate research assistants with a background in social marketing, split up and independently screened the 1316 titles and abstracts. They excluded 1061 studies either because they were unrelated to excessive sodium consumption (e.g., the studies pertained to undernutrition or to promoting the use of iodized salt), were an inappropriate study type (e.g., not peer-reviewed), or were not published in English, leaving 255 records. To avoid bias, all team members then discussed and agreed on the final set of studies; 158 were subsequently excluded by team consensus either because they did not meet at least one social marketing benchmark criteria (*n* = 70), because of the study type (*n* = 83), or publication language (*n* = 4), leaving 97 papers for full-text review. An additional 46 articles were excluded at full-text review, either because they were not classified as social marketing (*n* = 21), were a social marketing intervention still in progress on January 1, 2022 (*n* = 9), or were not an intervention (*n* = 16), leaving a final sample of 51 studies. Figure 1 summarizes the selection process.

### Data analysis

The first phase of data analysis consisted of creating an indexing matrix, drawing from matrix method frameworks (60,61,62,63). Specifically, we adapted the checklist developed by Kubacki et al. (2015) (Table II: Assessment of the use of Andreasen’s benchmark criteria in social marketing interventions) (64) as the foundation for a revised spreadsheet capturing social marketing interventions. This Microsoft Excel matrix consisted of rows for each of the 51 studies and columns for study name, authors, and publication date. Additional columns captured the social marketing benchmark criteria outlined in Table 1, adapted from Andreasen (2002) and French (2005), as well as subsequent applications and updates (25,34,64,65,66). We recorded and assessed each study/intervention to determine the following: its behavioral focus; whether its design was informed by formative research; whether it targeted a specific subgroup of the population (segmentation); whether it offered an exchange that was attractive or beneficial to the priority population or helped to counter competing behaviors; whether it considered alternative behaviors that the priority population might engage in instead of reducing salt/sodium consumption (competition); and whether it incorporated at least one of the 4Ps of the marketing mix (product, price, place, and promotion) (64). We further augmented the spreadsheet matrix with the PICO (TS) categories: population, intervention type, comparison, outcome, time, and setting (location). To avoid bias, researchers indexed all 51 studies independently, and then reconciled differences through consultation. The matrix also assessed the level of community-involvement (32)—that is, the extent to which the community participated in the design, implementation, or evaluation—and whether the intervention was integrated into broader policy and programming that incorporated stakeholder perspectives (67). Table 1 outlines the social marketing benchmark criteria and additional factors used to inform data extraction. Because we relied on each study’s self-reported effectiveness, effect measures varied from study to study. These measures are specified for each study in Table 2.

**Table 2:** Summary of behavior change (study effectiveness) findings by study.


STUDY	BEHAVIOR	BEHAVIOR CHANGE (STUDY EFFECTIVENESS)

An et al. (107)	Healthy food purchase among participants in one insurance program	2/3 of the difference in healthy food purchase between participants and non-participants was attributable to the program. Consumption of foods high in salt decreased by more than 20%

Anderson et al. (68)	Reduction in sodium intake through emphasis on spices and herbs	Mean 24-h urinary sodium excretion was significantly lower in the intervention group

Beer-Borst (39)	Reduction in sodium intake through workplace meal purchase intervention	The overall mean change in daily salt intake was –0.6 g (from 8.7 to 8.1 g, or 6.9%). Though the mean daily salt intake of women (7 g) was unaltered, the mean intake of men declined by –1.2 g (from 10.4 to 9.2 g). Baseline salt intake, sex, and waist-to-height ratio were significant predictors of salt reduction. The analysis also highlighted key determinants of low adoption and reach and effective program implementation in catering operations.

Bernabe-Ortiz et al. (105)	Promotion of a low sodium salt substitute by replacement with 25%KCl/75%NaCl at community and individual level (vendors, businesses, kitchens, bakeries, restaurants, and homes)	Of 2376 participants, an average reduction of 1.29 mmHg [95% confidence interval (95% CI) (–2.17, –0.41)] in systolic and 0.76 mmHg [95% CI (–1.39, –0.13)] in diastolic blood pressure. Participants without hypertension at baseline, in the time- and cluster-adjusted model – 51% [95% CI (29%, 66%)] reduced risk of developing hypertension compared with the control group. In 24-h urine samples: no difference in NA levels, mean difference 0.01; 95% CI (0.25, –0.23); K levels higher, mean difference 0.63; 95% CI (0.78, 0.47).

Bin Sunaid (40)	Reduction in sodium intake through government regulation, a marketing campaign, and educational materials in strategic locations such as restaurants and workplaces	Government compliance was moderate. Only 2% of manufacturers adopted front-of-pack nutrition labels. Data collected on 363 establishments revealed that 27% did not display caloric content, 25% had incomplete displays of caloric content, and 19% featured incorrect calculations. Of the 297 products assessed, 85% were compliant with the advised sodium limit of 1%; however, 25%–50% of pastries prepared with meat, hamburgers, and all processed meats were non-compliant with the government sodium recommendations.

Bouterakos (38)	Provision of salt-reduced meals in schools, and digital education of children to limit salt in the home	A total of 28 interviews were completed with 13 children, 11 parents, 3 principals, and 1 teacher. Children and adults self-reported dietary change and reduction of sodium intake. Participating children also showed improvements in self-reported salt-related knowledge and self-efficacy, and fewer children reported placing a salt shaker on the table.

Brown et al. (69)	Reduction in sodium intake	Church members in the intervention group showed a greater increase in fruit and vegetable intake than the control group [0.25 cups per day (95% confidence interval: 0.08, 0.42), *P* = 0.002], a greater decrease in sodium intake [–123.17 mg/day (–194.76, –51.59), *P* = 0.04], but no difference in moderate- or greater-intensity physical activity [–27 metabolic-equivalent minutes per week (–526, 471), *P* = 0.56].

Cateriano (28)	Understanding salt-related behaviors and salt use during cooking by observation through a journey-mapping process	In three stages of food shopping and cooking, 13 salt touchpoints were observed, with the highest number occurring during food preparation. No shopping lists were made, and though artificial ingredients containing salt were included in purchases, a variety of natural seasonings and spices were also purchased to enhance flavor. All participants tasted the food during preparation and flavored it with natural spices and herbs as well as salt. None of the participants put a salt shaker on the table at the time of serving the meal. Therefore, natural ingredients in cooking can be an opportunity to replace salt in the diet.

Cappuccio et al. (108)	Reduction in salt intake through community-based health-promotion intervention. The intervention included a health education program carried out by community health workers, with daily sessions for the first week and once weekly afterwards	There was no significant change in urinary sodium between groups, while the intervention group showed lower systolic and diastolic blood pressure

Chen et al. (94)	Reduction in salt intake	Participants in the intervention group (salt restriction spoon and health education) significantly decreased their salt consumption compared to the control group. Before the intervention, 26.1% of intervention group participants reported often or daily use of a salt-restriction-spoon, and 13.3% reported using it correctly. After the intervention, 67.3% of intervention group participants reported often or daily use of the spoon, and 37.3% reported correct use. This significantly higher use rate and correct use resulted in a daily salt reduction of 1.42 g, and a 24HUNa reduction of 34.84 mmol.

Chu et al. (135)	Reduction in sodium intake, measurement of effectiveness of a national population-level program	Health education activity attendance was 61.5% for general villagers and 92.3% for people with high risk of cardiovascular disease. The 18-month (*n* = 1903) 24-h mean urinary sodium excretion in the intervention arm was reduced by 5.5% (–14 mmol/day, 95% confidence interval –26 to –1; *P* = 0.03), while potassium excretion increased by 16% (+7 mmol/day, +4 to +10; *P* < 0.001), and the sodium to potassium ratio declined by 15% (–0.9, –1.2 to –0.5; *P* < 0.001).

Cornelio et al. (37)	Reduction in salt use during cooking through intervention aimed at improving self-efficacy and promoting behavior change	At 3-month follow-up, the intervention group improved significantly more than the control group regarding salt addition measures (*P*-values between 0.05 and 0.001) and psychosocial variables (all *P*-values ≤0.001). The reduction in 24-h urinary sodium excretion was not significant.

Cotter et al. (89)	A comparison of the effect of salt reduction education (THEOR) and a combination of education and practical gardening and cooking lessons (PRACT).	The group subjected to salt reduction education and practical activities saw a significant reduction in salt intake. At baseline, 139 students (76 girls and 63 boys) were eligible for the study, and showed an average 24-h UNa of 132 ± 43 mmol/24 h (mean salt intake of 7.8 ± 2.5 g/day) and a BP of 118/62 (13/9) mmHg. At the end of the study, BP decreased by 8.2/6.5 mmHg versus baseline in the control group (*n* = 31), by 3.8/0.6 mmHg in THEOR group (*n* = 43) and by 3.5/0.7 mmHg in the PRACT group (*n* = 53). Salt intake was reduced by 0.4 ± 2.4 g/day among the control group, by 0.6 ± 3.2 g/day in the THEOR group and by 1.1 ± 2.5 g/day in the PRACT group. Variation in salt intake was dependent on the group (χ, 9.982, *P* = 0.041). Salt intake was only significantly reduced in the PRACT group (1.1 g/day), and the percentage of children in this group who reduced salt intake by at least 1 g/day from baseline was significantly higher (50.9%) than in the other groups, (THEOR, 48.8% and CTR, 32.2%).

Cummings et al. (70)	Integration in sodium reduction strategies in food procurement and vending food venues	Three government departments adopted the new nutrition standards and requirements (including sodium limits and best practices such as the use of signage, pricing incentives and menu labeling), potentially affecting 100,000 meals sold daily in government venues.

Daivadanam (103)	Change in household dietary habits including reduction in sodium intake through procurement of fruits and vegetables (FV), substitution of fried foods with steamed or fruit snacks, changing the color of the food on the plate by increasing vegetables, increasing the display/accessibility of FV in homes a and decreasing the display of jams/pickles/fried snacks, buying local FV, and reallocating budgets to purchase more FV	Monthly household consumption of salt was reduced by 45% in the intervention arm compared to the control arm

Do et al. (100)	Reduction in salt intake intervention based on the COMBI framework. Activities included: mass media communication, communication in schools, community-level program and home visits.	Mean urinary sodium excretion fell significantly in the communities where the program was implemented. Mean sodium excretion in spot urine sampling fell significantly from 8.48 g/day at baseline to 8.05 g/day at follow-up (*P* = 0.001)

Eyles (87)	Improve cardiovascular health through lower salt food purchases mediated by the use of an app	A significant reduction in mean household purchases of salt (~0.7 g of salt per person per day) was noted during the 4-week intervention phase.

Fitzgerald (91)	Improve diet habits including salt reduction in the workplace through cafeteria catering and nutrition education for participants and catering staff in way that is cost effective for management. Interventions included menu modification, increase in fiber, price discounts, strategic positioning of food, and portion size control, individual nutrition consultants, and detailed nutrition information	This complex workplace dietary intervention combining nutrition education and system-level dietary modifications reduced employees’ intake of salt and saturated fat (significant reductions in on-duty intakes of total fat (–14.2 g/day, *p* ¼ 0.000), saturated fat (–7 g/day, *p* ¼ 0.000), salt (–1.4 g/day, *p* ¼ 0.000) and total sugars (–8.9 g/day, *p* ¼ 0.003), improved employees’ nutrition knowledge and decreased their body mass index in a cost effective way. The system-level and combined interventions had positive net benefits; the savings gained from reduced absenteeism were greater than the costs of the intervention. The system-level intervention had the highest net benefit: $53.56 per employee.

Fitzgerald (92)	Improved dietary habits at work, including salt reduction, extending to the employees’ off-duty eating habits through nutrition education (Education), environmental dietary modification (Environment), or both (Combined).	Improvements observed in employees’ dietary intakes at work also extended to their lives outside of work. Significant reductions in on-duty intakes of total fat (–14.2 g/day, *p* ¼ 0.000), saturated fat (–7 g/day, *p* ¼ 0.000), salt (–1.4 g/day, *p* ¼ 0.000) and total sugars (–8.9 g/day, *p* ¼ 0.003) were observed in the Combined and Environment groups [total fat (–11.4 g/day, *p* ¼ 0.017) and saturated fat (–8.8 g/day, *p* ¼ 0.000)]. In the Combined group, significant changes were also observed in off-duty intakes of total fat (–10.0 g/day, *p* ¼ 0.001), saturated fat (–4.2 g/day, *p* ¼ 0.001), salt (–0.7 g/day, *p* ¼ 0.020), and total sugars (–8.1 g/day, *p* ¼ 0.020).

Francis et al. (71)	Dietary habits including salt intake	The intervention group that received two dietitian-led education sessions at home consumed significantly less sodium than controls.

Fujiwara et al. (95)	Salt reduction to decrease urine albumin-creatinine ratio among albuminuria patients	Participants in the intervention group (those receiving family and community support to motivate behavior change) showed a significant reduction in albumin-creatinine ratio (ACR) from baseline. The intervention group had a lower ACR compared to the control group, although *P* = 0.007.

Gans et al. (113)	Teaching healthy salt and fat reduction techniques during cooking as part of a cook-off contest for home economics classes embedded in a wider healthy heart initiative	42 students with elevated blood cholesterol levels were invited to return for follow-up; 40 students (95%) returned. Mean change in blood cholesterol level from baseline to follow-up was 21.7 mg/dL (*P* < 0.0001). When expressed as a percentage of baseline, this represented a 10.7% average reduction in blood cholesterol (*P* < 0.0001).

Gonzales (86)	Adoption of national salt reduction strategy through the removal of access to sodium in restaurants, food service establishments and vendors	47 restaurants removed salt shakers, soy sauce, and finadene from their tables and establishments. At least 500 salt-reduction trifolds were distributed to participating restaurants, and 2000 additional pamphlets were made available for replenishing purposes during project monitoring to ensure sustainability.

Grimes (84)	Reduction in sodium intake through a consumer awareness campaign, and before and after surveys of parents/caregivers with children nationwide	There were limited changes in self-reported knowledge, attitudes, and behaviors among adults in this study. The strongest evidence of improvement related to the behaviors of children as reported by adults. The percentage of parents/caregivers who agreed that limiting salt in their child’s diet was important increased by 8% (*P* = 0.001), and this coincided with a 10% reduction in table-placed saltshakers, and a 9% reduction in salt added by children at the table (both *P* < 0.001).

Grunseit (81)	Reducing consumption of unhealthy takeaway foods, including those high in salt, through peer support therapy	Participants self-reported a reduced consumption of takeaway food by participating in four main activities: recasting reduction as saving money, making small changes, engaging in self-care, or goal-setting; adding practical changes to behavior in the way of planning, rule-making, or portion-adjustment; using external instruments such as the food environment and social support; and reconfiguring social events.

Ireland et al. (82)	Reduction in sodium intake	Participants receiving dietary education about choosing foods identified by a Tick symbol or Food Standards Australia and New Zealand’s low-salt guideline of 120 mg sodium/100 g food saw a significant reduction in urinary sodium excretion. Reported sodium intake (multiple-pass 24-h recall) significantly decreased only for the Food Standards group.

Johnston et al. (72)	Adoption of marketing strategies, taste demonstrations and media campaign by local grocery stores	Not reported

Jordan (76)	Salt reduction through meal procurement, cafeteria, restaurant and catering programs	Sodium content of targeted foods or meals decreased by 261 mg (from 946 mg at baseline to 685 mg at final follow-up) in the 12 food service settings that submitted data.

Kim (99)	Salt reduction through use of a mobile health app to encourage behavioral changes in individuals with metabolic disorders	Preference for a low-sodium diet, reading nutritional facts, having breakfast, and performing moderate physical activity significantly increased in the mHealth intervention group (IG) (app users) as compared to the conventional (CG) health center users. At baseline, the practice of reading nutritional facts was significantly lower in IG (15.81%) than in CG (25.73%) (*P* = 0.0007) after 24 weeks it was significantly higher in for IG (46.10%) as compared to CG (28.64%, *P* < 0.0001). Within each group, low salt preference and label-reading significantly rose in IG over 24 weeks (*P* < 0.0001 and *P* = 0.0006, respectively), with no significant change in CG.

Klassen (77)	Awareness of the link between dietary sodium, hypertension and stroke among a hard-to-reach priority population (20- to 40-year-old Black males) through public education campaigns and street intercept surveys	30% of post-campaign respondents reported familiarity with key campaign content compared to only 17% of pre-campaign respondents; 17% post-campaign respondents provided accurate recall; 41% recalled stroke relationship to salt. Those respondents who remembered the key phrase ‘Mom Says’ in one of the campaigns were 95% more likely to remember the connection between salt and stroke.

Land et al. (83)	Reduction in salt intake	Mean salt intake (measured through 24-h urinary excretion) significantly decreased, while knowledge of recommendations about salt reduction and strategies to reduce salt improved following implementation of a multi-faceted community-based program. However, the proportion of people who checked food labels and avoided processed food decreased. Overall, a 10% reduction in salt consumption was observed in the community.

Layeghiasl (29)	Reduction of salt intake in 25- to 50-year-olds	A social marketing program featuring educational materials, classes, phone counseling, and brief interventions by health personnel produced a significant reduction in salt intake (3.01 ± 2.38 g/day), and a significant change in knowledge (mean ± standard deviation of change = 2.58 ± 1.3, *P* = 0.001), attitude (mean ± standard deviation of change = 1.68 ± 4.03, *P* = 0.001) and practices (mean ± standard deviation of change = 3.37 ± 2.92, *P* = 0.001) in the intervention group, while this did not change in the control group.

Lee Kwan et al. (74)	Customer reach of Baltimore Healthy Carry-outs and purchase of healthier products	Intervention included improvements to menu boards and labeling to promote healthier items, introduction of healthier sides and beverages as well as affordable healthier combo meals. Purchases of healthier foods increased by almost 40% compared to baseline at intervention carry-outs.

Livingston et al. (93)	Reduce intake of discretionary foods through personalized (phenotypic and genotypic) nutrition advice	Three levels of intervention produced different results: for L1 (diet and physical activity), L2 (diet and activity+ personalized phenotypic feedback based on nutrient and metabolic biomarkers), and L3 (diet and activity + phenotypic + genotypic feedback based on variants in nutrient-responsive genes). L2 and L3 randomizations resulted in greater reductions in the percentage of energy, total fat, saturated fat, and salt consumed through discretionary foods, with a 0.48 ± 0.17 difference in salt intake between the control group and the intervention group.

Long (78)	Salt reduction through community meals program	Across three programs, the mean amount of sodium served per diner from baseline to Year 1 follow-up decreased from 1443 to 864 mg (–40%). The mean amount of sodium served per diner in Year 2 was 920 mg, which was more than the 864 mg observed in Year 1 (+6%) but less than baseline (–36%). At the Year 3 follow-up, the mean amount of sodium served per diner was 944 mg, which was more than Year 2 but less than baseline (–35%)

Ma (79)	Promote physical activity and reduce dietary sodium intake	An educational intervention culturally tailored to Filipino Americans produced a non-statistically significant decrease in urine sodium, a blood pressure reduction of 12.6 mmHg, and diastolic pressure decrease of 3.8 mmHg in the intervention group

Ma (102)	Reduce salt intake in children through social group association	Salt reduction score (SRB) = Answer to three questions on questionnaire: (1) high-salt snack frequency; (2) high-salt pickle frequency; (3) family salt reduction (y/n). A 1-unit increase in SRB was associated with a 0.31 g/day greater reduction in salt intake during the trial (95% CI 0.06 to 0.57, *P* = 0.016). The more family members a child had who did not support salt reduction, the lower the SRB. Children with more friends had higher SRB scores (all *p* < 0.05). Children whose teachers attended the intervention had higher SRB scores (*P* = 0.043)

Nader (73)	Reduction in salt intake through year-long family-focused intervention	Anglo-American families in the study reported a lower sodium intake than Mexican-American families. Overall, the 3-day salt score significantly decreased in both groups.

Perlmutter (75)	Acceptability of low sodium entrees in a workplace cafeteria	No significant difference in sales was observed after the introduction of modified entrees, and no significant changes were observed in overall acceptability. When entrees were advertised as lower in fat and sodium, consumers reported higher acceptability.

Ponce-Lucero (106)	Identification of population-level salt reduction social marketing campaign audience	The study conducted formative research to inform the development of an intervention

Savedra et al. (104)	Sales of lower sodium breads	Introduction of lower sodium breads did not change sales, and bread samples prepared with less sodium were not discernible from regular bread

Sakaguchi (98)	Sodium reduction through a one-year work-related healthy lunch and nutrition education program	A significant decrease in urine sodium (a –4.6 g change from 14.2 to 9.6 g; 95% CI: –7.1, –2.1) was observed in the workers who consumed healthy lunches, compared to no significant change in the group that did not consume healthy lunches. The difference between the intervention and control groups was not significant.

Sosa (80)	Sodium reduction through a work-related cafeteria and congregate meal programs	All worksites improved on five out of seven sodium practices. At follow-up, when compared to baseline, more worksites reported using recipes (75% vs. 50%), measuring salt while cooking (100% vs. 75%), and reducing salt by offering smaller portion sizes (88% vs. 75%). For congregate meal programs, six out of seven sodium practices stayed the same from baseline to follow-up.

Talaei et al. (110)	Adoption of modified (lower sodium, higher in fiber) recipes for bread by bakeries (healthy bread)	Number of bakeries producing HB increased from 1 to 402 (41% of bakeries in intervention area) after 6 years. People who lived in the intervention area consumed significantly more whole grain bread than people in a control city.

Trieu (88)	Reduction in salt intake through awareness campaigns, community mobilization, and policy/environmental changes	The outcome evaluation found that while there were significant improvements in knowledge and self-reported behavior (intermediate outcomes), there were no changes in mean salt intake (7.3 g/day in 2013 vs. 7.5 g/day in 2015; *P* = 0.588)

van’t Riet et al. (90)	Reduction in salt intake following messaging promoting a low-salt diet	Overall there was no difference between participants based on self-efficacy level or frame of messaging received (gain vs. loss-framed messaging). For participants in the high self-efficacy condition, loss-framed messages were more effective than gain-framed ones in influencing participants’ intention to reduce salt intake.

Vaughn (27)	Improve diet (including reduced salt intake) and increase physical activity through childcare-based intervention	No significant changes were noted in any of the outcome measures except for small improvements in children’s sodium reduction (mean change = 0.52, *P* = 0.029).

Webster (85)	Reduce salt intake nationwide through voluntary food industry salt reduction, strategic health communication, and a hospital meals program	The evaluation showed a 1.4 g/day drop in salt intake from the 11.7 g/day at baseline, however this change was not statistically significant.

Wentzel (109)	Reduce population-level discretionary salt intake through a mass media campaign	Most of the indicators of knowledge, attitudes, and behavior change showed a significant move toward considering or initiating reduced salt consumption. Post-intervention, significantly more participants took steps to control salt intake (38% increased to 59.5%, *P* < 0.0001) by avoiding adding salt during cooking and at the table.

Wong (101)	Lifestyle intervention including the reduction of salt intake	Significant improvements in moderate-intensity physical activity (PA), vigorous-intensity PA, and total PA (*P* < 0.001), increased intake frequency of fruit and vegetables (*P* = 0.049), a reduction in salt and sugary beverage intake (*P* ≤ 0.042), and reductions in systolic blood pressure (BP; –3.68 mmHg), diastolic BP (–3.54 mmHg), and percentage body fat (–2.13%; *P* ≤ 0.020) when compared with the control group.

Yang, 2021 (97)	Reduce salt intake through social media campaign	Post-intervention, the salt-related knowledge score was relatively lower, while the salt reduction behavior score and high-salt intake behavior score were relatively higher. The percentage of participants who used salt measuring spoons, asked restaurants to use less salt, read the sodium content of foods, chose foods with low sodium content, and regularly used low-sodium salt increased significantly post-intervention (all *P*-values <0.05).


## Results

We included a total of 51 studies in this review. Of these, 21 studies spanned the years 1989 through 2016, and 28 were conducted between 2017 and 2022. This represents a substantial increase in social marketing scholarship related to salt/sodium reduction over the past 10 years.

### Variation by geographic location

**Figure 2:**
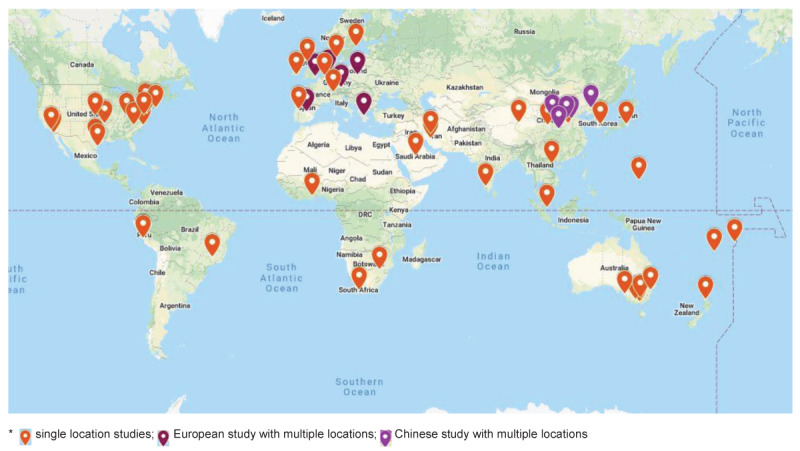
Location of studies 1989–2021.

A review of study locations indicated clusters centered in Africa, the Americas, Asia, Europe, the Eastern Mediterranean, and the Western Pacific, and these were mapped using Google Maps (Figure 2). The highest concentration of studies occurred in the Western Hemisphere in the United States (*n* = 14; 27,68,69,70,71,72,73,74,75,76,77,78,79,80), and in the Eastern Hemisphere in Australia (*n* = 5; 38,81,82,83,84), and Oceania (*n* = 4; 85,86,87,88). Multiple interventions also took place in Europe (*n* = 6; 39,89,90,91,92,93), northern Asia (*n* = 6; 94,95,96,97,98,99), Southeast Asia (*n* = 4; 100,101,102,103), and in South America (*n* = 5; 28, 37,104,105,106). A few interventions (*n* = 3) were conducted in Africa (107,108,109), and in the Middle East (*n* = 3; 29,40,110).

### Variation by objectives

Most of the reviewed studies aimed to address individual-level change related to salt/sodium consumption (27,37,39,40,62,68,70,72,75,79,81,82,83,88,89,90,93,94,95,96,99,100,101,102,103,104,105,106,108), either by measuring actual intake (self-reported or observation) or by using 24-h urinary sodium excretion as a proxy. In some cases, intention to reduce intake (90) and acceptability of low-sodium recipes (39,76,78,79,80,84,109) were the focus. A few studies aimed to promote healthy eating more broadly (28,40,71,74,81,86,92,93,107,109,110), with some focused on healthy lifestyle promotion, including but not limited to diet (69,73,79,86,92,93,96,97,99,101,103). Many studies had an upstream focus (38,39,40,70,76,78,80,81,85,88,91,92,98,105), seeking to lower salt/sodium consumption by intervening in the food procurement process—through meal programs, catering, or cafeteria service at the county, work, school, or hospital level. Some studies attempted to reduce salt intake nationwide by targeting restaurants, either in part or as the primary target (40,76,78,83,86,88,102). Several studies examined whether peer influence and social group norms could effectively promote salt/sodium reduction (81,88,97,102). A few compared the effectiveness of two or more interventions (82,89,90,111), and some had secondary objectives to evaluate the effectiveness of salt reduction campaign messaging (38,39,97,100,112). For example, the Beer-Borst et al. (39) intervention had a primary objective of educating catering staff on how to prepare healthier food for a workplace cafeteria, thereby reducing workers’ salt intake, and a secondary objective of enhancing the marketing, messaging, and placement of healthier food so that it would be both pleasing and easy for workers to make healthier choices once they entered the cafeteria lines (39).

### Variation by study designs

To achieve their objectives, some studies used experimental designs—such as randomized controlled trials (27,37,68,69,71,73,74,82,87,88,89,90,91,92,94,96,101,102,103,105,108), quasi-experimental (29,39,75,83,95,98,104,110), and observational designs (28,72,100,107)—while others employed qualitative methods (29,38,39,70,77,81,96,97,105,106,113). A few studies explicitly incorporated theories, theoretical constructs, or conceptual frameworks in the development of their interventions, such as: self-efficacy and self-determination (69,90); social cognitive theory (27,73,101); the social ecological model (27,81,88); the communication for behavioral impact framework (83,100); the community-based participatory research framework (86); the theory of reasoned action (109); behavioral change theory (38,85); social network theory (102); exchange theory (27); the transtheoretical model (37); the formative process evaluation framework (74,85,88,96); the sequential exploratory method (106); and the health belief model (94). Many studies specifically referred to social marketing as the guiding conceptual framework for their design (27,28,29,40,72,77,79,84,85,88,92,100,101,105,106,114).

### Variation by priority populations and segmentation

A population of interest was identified in most studies, although the process through which segmentation was applied was often not clarified. While demographic and geographic segmentation was the preferred method, some studies incorporated behavioral and psychographic characteristics, as defined by social marketing principles (115). For example, some studies targeted school-aged children living in rural communities, along with their teachers and/or parents (27,28,38,73,78,80,84,89,100,102,106,113,116), while others focused on workers who used workplace cafeterias, and the staff who prepared the cafeteria food (39,75,76,80,91,92,98). Additionally, some studies focused on owners and staff at food establishments such as bakeries and grocery stores (72,104,105,110), local church charities (69,88), and restaurants (40,70,75,76,78,80,83,86,88,98,105). Some studies incorporated race or ethnicity, geographic location, or certain lifestyle factors (40,78,79,81,85,86,101,103,109). Another important characteristic used for segmentation was adults within specific age groups, with defined risk factors, interested in adopting a healthier lifestyle (37,69,71,76,78,95,100,108). For instance, Cornelio (37) specifically targeted Brazilian women with hypertension, Brown et al. (69) focused on non-pregnant LatinX and European women in Corpus Christi interested in reducing stroke risk factors, and Francis et al. (71) engaged older women (aged 54–83) in cardiovascular disease (CVD) education. Later studies, such as Do (76), Jordon et al. (78), Bernabe-Ortiz et al. (100), and Long et al. (105), targeted entire communities with the goal of reducing salt intake more generally. Some studies combined community-wide salt reduction strategies to reduce specific risk factors or diseases, such as *albuminuria* and high blood pressure (95,105,108).

### Variation by type of formative research

Most studies used some degree of formative research with the priority population. Literature reviews often marked the start of the formative research process (8,27,29,46,51,67,77,85,93,98,102,105,106,117), followed by primary data collection through surveys (29,77,79,88,98,102,103,105,106), focus groups (29,74,77,78,79,85,88,106), interviews (74,80,88,106), direct observation (28,74,105,113) and journey maps (28).

### Variation by mode of exchange

In social marketing, ‘exchange’ refers to the provision of something of benefit to counteract a competing behavior currently exhibited by the priority population. In the case of salt reduction initiatives, salt quantities needed to be lowered or replaced with something of equal or greater value to participants. A potential risk of bias here is that most studies found this exchange to be a particularly difficult process, primarily due to taste preferences. Nevertheless, successful exchanges did occur. Some studies offered tangible exchanges, such as a salt substitutes or alternative spices (29,37,68,80,83,96,100,105,109); others offered monetary incentives, such as discounts for purchasing low-salt meals or free meals made with low-salt recipes (40,72,76,78,80,91,92,98); others offered electronic exchanges in the form of free apps, software, or self-regulation tools to inform the use of salt-containing foods (38,76,79,83,87,99,109); and some offered sociological exchange, such as peer support, culturally appropriate workshops, or in the case of school interventions, recognition in the form of trophies, stickers, and certificates (37,38,78,80,88,89,100,102,106,108,113).

### Variation by competition

Most papers accounted for competing behaviors that the priority population might engage in. That said, a risk of bias—however negligible—did exist for this synthesis, because the term ‘competing behavior’ was not always explicitly mentioned in the studies. Some successful applications of competition were found in workplace-based interventions and hospital cafeterias (39,72,76,80,91,92,98). In these studies, cafeterias used pricing strategies to overcome competition; they offered substantially discounted salt-reduced meals alongside regularly priced non-salt-reduced meals, encouraging participants to overcome the competing behavior. When faced with the choice of spending half as much on a meal at lunch, patrons were enticed to choose the healthy option over more expensive, salt-riddled options (40,72,76,80,91,92,98). Some school-level interventions used competition explicitly, with classrooms and students vying against each other to overcome the competing behavior faster than their peers. This was achieved through the infusion of ‘price’ and ‘product’ concepts, such as offering tangible rewards like balloons, stickers, gym bags, t-shirts, chef hats, and aprons to the classrooms with the least competing behaviors (27,38,113).

### Variation by intervention design/marketing mix

**Figure 3:**
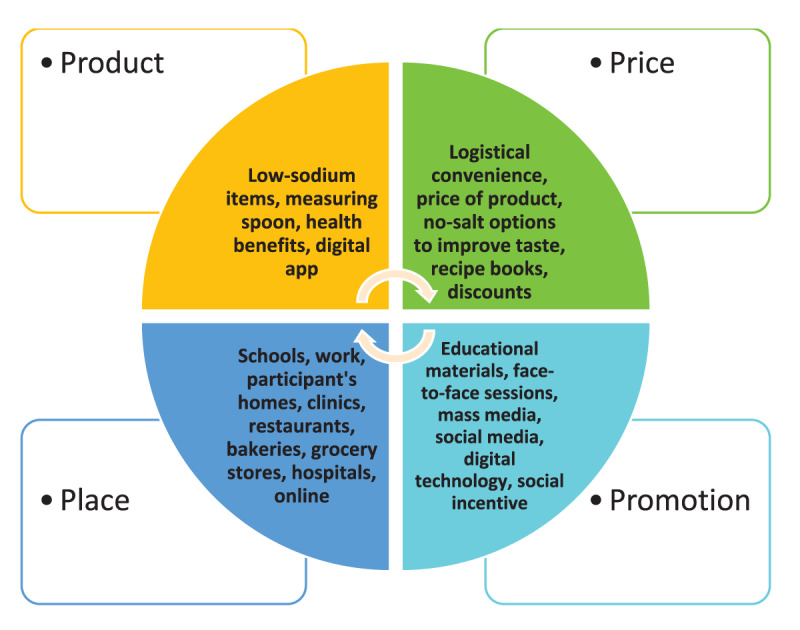
Marketing mix: product, price, place, and promotion strategies.

All studies implemented some form of the marketing mix, either upstream or downstream. Upstream interventions included changes in food procurement procedures, the education of county-level stakeholders, and the development of sodium-level recommendations (29,38,39,40,70,76,78,80,81,85,88,91,92,96,98,101,105,109). Downstream interventions incorporated elements of the marketing mix, specifically the four Ps (product, pricing, placement, and promotion). Promotion was the most explicitly identified component when describing the interventions. Figure 3 summarizes the main results related to the 4Ps.

Interventions included the use of a measuring spoon to limit salt use (94), the promotion of low-sodium products through cash incentives or labeling (27,40,72,74,75,76,80,82,83,85,104,107,110), the distribution of recipes and cookbooks promoting low-sodium cooking (76,78,79,80,84,109,113), discounted pricing on low-sodium food and cafeteria meals (40,72,76,80,91,92,98), the promotion of salt alternatives such as spices and herbs (27,29,38,76,80,83,85,86,87,88,91,92,97,101,105,106,109), information campaigns on the risks of excessive sodium consumption (e.g., educational sessions, materials, and advertising; 37,38), hands-on activities such as cooking classes and gardening (27,68,79,101), and even digital apps and tools (40,79,87,97,99).

### Beyond the intervention: Community involvement

Community-involvement emerged as a key component in many studies (Table 2). In some cases, interventions engaged families, workplaces, schools, and neighbors of the priority population to promote long-term impact (27,28,29,38,39,73,76,77,78,80,81,84,86,88,91,92,93,95,96,98,99,101,102,103,105,109,118). A notable example is Chu et al. (96), a clustered randomized controlled trial involving three million participants in 120 rural villages in Northern China, which included a mixed methods process evaluation. Quantitative data were collected from 120 record logs maintained by project officers and assistants, while qualitative data were gathered through purposeful, in-depth interviews with 48 key stakeholders in 10 intervention villages. These stakeholders included provincial managers, doctors, and participants. At the regional level, government officials and health educators implemented chronic disease policies that supported the distribution of communication materials and free pre-packaged low-sodium salt substitutes in local stores. This initiative’s success depended on the grassroots support of village community members, including administrative staff, community elders who spread the word, children who brought adults on board, and village medical personnel who lent the program credibility. Over 18 months, the intervention reduced sodium levels by 5.5% (96). This example highlights how integrating community involvement with multi-level structural change can be critical to the success and sustainability of long-term interventions. Many other studies also demonstrated the importance of community and stakeholder engagement for ensuring sustainable results (38,76,78,79,84,91,92,101,105). In fact, studies often combined community-based implementation with local, regional, and national governmental policy support (38,40,70,76,80,83,84,85,86,96,105,108,109). Community based interventions were also successfully paired with schools (38,78,100), workplaces (76,91,92), businesses (74,83,105), and participant homes (38,103), and sometimes included clinical elements as well (29,81,83,99,109).

### Variation by social marketing benchmark criteria use

Overall, there has been a marked increase in the integration of social marketing concepts in the design, implementation, and evaluation of salt/sodium reduction interventions globally. Between 2017 and 2022, 30 studies were published on salt reduction using social marketing techniques; of these, 13 applied all 8 social marketing benchmark criteria, while only 1 (97) used fewer than 6 criteria (see Table 3). As shown in Table 3, more recent studies tend to be multi-level and to integrate broader policy and program-level components. More recent studies also demonstrate closer adherence to social marketing benchmark criteria in both their design and implementation. Notably, interventions that were the most integrated and applied the greatest number of social marketing components also appeared to be the most successful in reducing salt consumption (29,37,38,72,76,78,79,81,82,84,91,92,96,101,104,105,109,110,113). Conversely, programs that failed to achieve significant results often omitted several social marketing benchmark criteria. This finding aligns with previous social marketing literature (51,65,115).

**Table 3:** Social marketing benchmark criteria checklist for included studies.


STUDY	YEAR	BEHAVIORAL FOCUS	SEGMENTATION	FORMATIVE RESEARCH	EXCHANGE	COMPETITION	MARKETING MIX	COMMUNITY INVOLVEMENT	INTEGRATION

Nader et al.	1989								

Gans et al.	1990								

Perlmutter et al.	1997								

Cappuccio et al.	2006								

Francis et al.	2009								

Fujiwara et al.	2010								

Ireland et al.	2010								

van‘t Riet et al.	2010								

Chen et al.	2013								

Cotter et al.	2013								

Lee Kwan et al.	2013								

Talaei et al.	2013								

Cummings et al.	2014								

Johnston et al.	2014								

Anderson et al.	2015								

Brown et al.	2015								

Saavedra et al.	2015								

Cornelio et al.	2016								

Do et al.	2016								

Land et al.	2016								

An et al.	2017								

Eyles et al.	2017								

Wentzel et al.	2017								

Daivadanam et al.	2018								

Fitzgerald et al.	2018								

Trieu et al.	2018								

Webster et al.	2018								

Beer-Borst et al.	2019								

Grunseit et al.	2019								

Kim et al.	2019								

Ma et al.	2019								

Sosa et al.	2019								

Gonzales et al.	2020								

Ponce-Lucero et al.	2020								

Klassen et al.	2020								

Bouterakos et al.	2020								

Fitzgerald et al.	2020								

Grimes et al.	2020								

Jordon et al.	2020								

Layeghiasl et al.	2020								

Bernabe-Ortizet al.	2020								

Yang et al.	2021								

Bin Sunaidet al.	2021								

Livingstone et al.	2021								

Cateriano et al.	2021								

Sakaguchi et al.	2021								

Vaughn et al.	2021								

Chu et al.	2021								

Longet al.	2021								

Ma et al.	2021								

Wong et al.	2021								


Grey: identified or partially identified; White: missing or not explicitly reported.

### Impact

Some studies employed quantitative measurements to gauge effectiveness, but all incorporated qualitative methods at some point—through surveys, interviews, focus groups, or other post-intervention stakeholder evaluation tools. Table 2 summarizes the reported impact of each intervention in achieving its desired behavior change. Overall, the reviewed interventions were heterogenous in their effectiveness, ranging from minimally to highly effective. Those that were less effective often excluded several key components of the social marketing benchmark criteria, including the marketing mix. In contrast, more effective interventions typically combined both upstream and downstream approaches, practical and educational strategies, longer implementation periods, and tailored the intervention to the culture of the priority audience. The most effective interventions were national policy-driven initiatives, community-based programs, and efforts supported by a broad base of stakeholders—including marketing, distribution, and service industries—that reinforced local grassroots efforts. In these cases, government and organizational support proved critical to success.

## Discussion

Literature on the application of social marketing to the reduction of salt/sodium intake at a global level was heterogeneous in terms of study designs, segmentation approaches, translation of the marketing mix into practice, and opportunities for sustainability. Several themes and trends emerged.

### Emerging themes and trends

A common theme with implications for practitioners was the lack of detail about how interventions were developed, particularly regarding formative research, the selection of priority populations, and the identification of marketing mix components (111,119). Early studies tended to mention promotional activities and educational sessions but provided little detail on their specific content. In contrast, later studies were more explicit, noting stakeholder involvement in identifying target populations for campaigns and interventions, and detailing evaluation processes (78,84,86,96,105). This increased transparency around audience segmentation and marketing mix development helps to inform future interventions and support replication (111,112,116,119,120).

One emerging trend is the increased use of social marketing as a guiding framework. More recent studies explicitly named social marketing, identified their use of exchange theory, the marketing mix, integration, and community involvement, and positioned these features as critical to their intervention’s effectiveness. As social marketing continues to be successfully applied in salt reduction efforts, the data support encouraging public health practitioners to apply and explicitly label its frameworks in public health and clinical practice (114,120), and to continually refresh its application by using it in new ways (121,122).

This marked increase in the application of social marketing to salt-reduction initiatives may be partly due to the release of the WHO’s *SHAKE the Salt Habit* in 2016, which provided guidance for member states on developing, implementing, and monitoring national sodium-reduction programs (123). Since the release of this publication, there has been a gradual growth in both the number of studies and the use of social marketing principles and benchmark criteria (see Table 3).

Replication and adaptation are also notable trends. For example, studies from Australia’s Victorian Salt Reduction Partnership project and studies across Oceania (Samoa, Guam, Fiji, and Singapore) all seem to have built on one another’s lessons (38,81,84,85,88,101). Overall, the evidence shows that social marketing interventions are effective at reducing salt intake—measured through self-reported behaviors or urinary excretion—and long-term assessment supports their sustainability (21,47,93,119).

A fourth trend is the shift toward upstream and multi-level approaches (24). While early research focused mainly on downstream efforts, studies published in the last five years have engaged policymakers, food manufacturers, public health officials, researchers, administrators, and producers, among others. Social marketing approaches emphasize the importance of engaging a broad base of individuals in the planning and implementation stages, recognizing them as vital to the success of any intervention. The cost effectiveness of such interventions is discussed at length in some studies (13,15,16,17,46,85,88,91). For example, workplace-based interventions emphasized the importance of working with and gaining the support of employers (39,80,91,92,98), school-based interventions focused on teacher and principal support (38,102,113), and hospital-based interventions encouraged engagement with cafeteria staff and hospital administrators (37,70,76,85). Interventions targeting multiple levels of the social ecology are consistently more effective (44,105,124,125,126), and have been explicitly encouraged by the WHO in several publications on salt-reduction (2,4,41,42,44,45,47,123,127,128,129,130,131).

### Localized and personalized interventions

As we rely more on schools, universities, and workplaces for daily meals, complex food-service interventions will become increasingly important for delivering nutritional education and dietary strategies. In this review, place-based interventions targeting schools, workplaces, recreation centers, and hospitals were the most successful, but only when healthy meals were offered at a discount or made the default option.

The effectiveness, sustainability, and acceptance of place-based interventions depended heavily on environmental and structural support. While discounting healthy meals increased their uptake, implementation required management buy-in and financial investment. Employers and administrators were more likely to support interventions once they recognized the long-term benefits, such as reduced health care costs and improved productivity. Studies consistently showed that management and administrative support were vital for intervention success (96,98,99). Where such support was lacking, interventions were generally ineffective (85).

Regarding communication, in-person visits and household-level interventions achieved stronger results than generic public information campaigns in the studies analyzed. Mobile applications—particularly those that enabled barcode scanning, access to nutritional information, and electronic recordkeeping—were the most successful, and outperformed websites, recipe books (76,78,79,80,84,109,113), WeChat (97), other mobile applications (99), and social media (97). This is in keeping with the findings of other studies that employed barcode-scanning mobile applications for dietary interventions (132). National media campaigns were most impactful when coupled with more localized and targeted interventions. Without follow-up social marketing activities, large-scale campaigns were not successful (40). Conversely, social marketing interventions that were not tied to higher-level state or federal campaigns with widespread messaging were also not sustainable (85).

## Limitations

This review has several limitations. First, the search strategy may have missed interventions that applied social marketing principles but did not label them as such. Second, although all of the studies considered were grounded in behavioral, methodological, and theoretical frameworks, there was variance in methodological rigor, with implementation science and evaluation processes becoming more precise over time. Applying a fixed scoring framework may have missed or under-valued context-specific adaptations or innovations. Third, publication and time-lag bias may have prevented us from identifying all projects in which social marketing was successfully applied to reduce salt/sodium consumption. Studies written up post-COVID-19 or published after 2022 were excluded (e.g., 26,30,49,133). Some multi-year social marketing studies were only partially captured in this review, as final evaluation and papers were pending (28,84,106,117,134). Fourth, this review only included studies published in English from four major electronic databases. Since 2021, advances in AI-assisted translation and screening tools have made it more feasible to include studies in additional languages. Finally, clusters of national social-marketing salt-reduction initiatives have emerged in regions such as Southeast Asia and the Middle East, but details were often unavailable at the time of our review. For example, Ghimire et al. (134) reported on programs at various stages of implementation in Afghanistan, Bangladesh, Bhutan, India, Nepal, Pakistan, Sri Lanka, and the Maldives, but only one Indian study (112) was included in this review. Similarly, Al-Jawaldeh et al. (117) described initiatives in the Middle East, of which only Iran and Saudi Arabia’s studies were captured in this review (29,40,110).

## Implications for Research and Practice

This review found that salt-reduction programs were most successful when they employed a greater number of social marketing benchmark criteria; interventions fell short when key components were missing. Community-based, localized, and culturally grounded tactics—such as engaging elders, doctors, and community health centers to spread intervention messaging, alongside children and youth volunteers working to sustain behavior change—proved especially effective. Likewise, hospital-, workplace-, and school-based interventions were most successful when they were supported by management, and only gained population-level traction when they were community-supported, culturally informed, and used local volunteers. Across all settings, multi-level interventions that addressed upstream policy and ensured downstream community engagement were most effective. National campaigns were only successful when coupled with midstream and local logistical support in both their planning and implementation.

The broader implications of these findings extend far beyond salt reduction and are transferable to other behavior change initiatives. They reveal vital lessons about social marketing over the past 40 years, particularly its ability to drive sustainable paradigm shifts and connect the efforts of individuals, communities, and governments for the greater social good.

Future efforts should focus on increasing awareness of social marketing and its application to global issues, promoting consistent terminology, and providing detailed accounts of how interventions are developed during the design phase. This would improve knowledge-sharing and support the translation of findings from study settings to real-world contexts. Incorporating long-term assessments and systems for continuous improvement would also help to promote intervention sustainability. But given the rising global demand for effective behavior change initiatives—particularly ones that work at population and structural levels—social marketing is well-positioned to be among the most impactful frameworks. In fact, any nutritional, lifestyle, or public health intervention that seeks to create sustainable behavior change could learn from these findings and adapt them to achieve greater reach and effectiveness.

## Data Accessibility Statement

The datasets used or analyzed during the current study are available from the corresponding author on reasonable request.

## Additional Files

The additional files for this article can be found as follows:

10.5334/gh.1478.s1 Appendix A. PRISMA Checklist.

10.5334/gh.1478.s2 Supplement 1. Full Search Strategy.
