# Supplementary material for: Systematic Review of Social Marketing as a Behavior Change Agent in Salt Reduction
Source: Glob Heart. 2025 Oct 24;20(1):100. doi: 10.5334/gh.1478 (PMC12551645; doi:10.5334/gh.1478)
Supplement: Supplement 1. — Full Search Strategy. [file gh-20-1-1478-s2.pdf]

# Supplement 1

## Full Search Strategy

### Search string

| | |
|---|---|
| Sodium consumption | Salt OR Sodium OR Sodium Chloride[MeSH Terms] OR Dietary Sodium [MeSH Terms] |
| Reduction | Excessive OR Reduc* OR Decreas* OR Restric* |
| Social marketing | “Social marketing” OR "Health communication” OR “Health promotion” OR Social marketing [MeSH Terms] OR “Marketing research” OR “Audience research” OR “Health Behavior” or “Behavior change communication” |

### PubMed Example

| |
|---|
| ((((((Sodium Chloride[MeSH Terms]) OR (Dietary Sodium[MeSH Terms]) OR Salt) OR Sodium)) AND ((Excessive OR Reduc* OR Decreas* OR Restric*))) AND ((((Social marketing[MeSH Terms]) OR (“Social marketing” OR "Health communication” OR “Health promotion” OR “Marketing research” OR “Audience research” OR “Health Behavior” OR “Behavior change communication”)))))) |
